# Genetic Analysis of Novel Fertility Restoration Genes (*qRf3* and *qRf6*) in Dongxiang Wild Rice Using GradedPool-Seq Mapping and QTL-Seq Correlation Analysis

**DOI:** 10.3390/ijms241914832

**Published:** 2023-10-02

**Authors:** Wenshan Cai, Wanlin Li, Liuying Duan, Yaling Chen, Fantao Zhang, Biaolin Hu, Jiankun Xie

**Affiliations:** 1 Laboratory of Plant Genetic Improvement and Biotechnology, Jiangxi Normal University, Nanchang 330200, China; cws0297@163.com (W.C.); wanlinli2020@163.com (W.L.); 17779145535@163.com (L.D.); yaqing620@163.com (Y.C.); 004768@jxnu.edu.cn (F.Z.); 2 Rice Research Institute, National Engineering Laboratory for Rice (Nanchang), Jiangxi Academy of Agricultural Sciences, Nanchang 330200, China

**Keywords:** fertility restoration, Dongxiang wild rice, QTL, GradedPool-Seq

## Abstract

The improvement of grain yield, quality, and resistance can be achieved through the utilization of heterosis. The combination of cytoplasmic male sterility (CMS) and fertility restoration (*Rf*) gene(s) greatly facilitates the commercial development of three-line hybrid rice based on heterosis. The basis for investigating the relationship between CMS and *Rf* genes lies in the rapid localization of wild rice fertility restoration genes. A set of the BC_4_F_5_ population derived from interspecific crosses between Xieqingzao B (XB) and the BC_1_F_9_ XB//Dongxiang wild rice (DWR)/XB line L5339 was used to detect quantitative trait loci (QTL) for fertility restoration. The population was then crossed with two male sterile lines, Zhong9A (Z9A) and DongB11A (DB11A), in order to generate a testcrossing population for investigating spikelet fertility. Based on the linkage mapping, seven QTLs were detected on chromosomes 1, 3, 5, 6, 8, and 10, explaining 2.76 to 12.46% of the phenotypic variation. Of them, two novel fertility restoration QTLs, *qRf3* and *qRf6*, can restore fertility of the CMS-DWR line DB11A by 16.56% and 15.12%, respectively. By employing joint QTL-seq and GradedPool-Seq methods, two novel *Rf* QTLs for DB11A, *qRf3* and *qRf6*, were identified at the physical locations of 10,900,001–11,700,000 bp and 28,016,785–31,247,556 bp, respectively. These findings are useful for exploring the natural variations of *Rf* genes in rice. Therefore, rice’s new genetic resources for the selection and breeding of rice restorer lines provide promising candidates for QTL fine localization and clarification.

## 1. Introduction

Male sterility (MS) is a widespread occurrence in higher plants, encompassing cytoplasmic male sterility (CMS) and genic male sterility (GMS), which is a pollen abortion-induced maternally inherited phenomenon causing fertilization and fruiting abnormalities. CMS is a maternally inherited phenomenon arising from pollen failure, resulting in abnormal fertilization and fertility. It is co-regulated by mitochondrial sterility genes and restorer of fertility (*Rf*) genes [1,2]. CMS and *Rf* function predominantly through cytoplasmic mitochondrial genes inducing male sterility, while *Rf* governs transcription or expression of sterility genes in mitochondria, thereby restoring fertility. The utilization and selection of *Rf* gene and breeding of restorer lines are pivotal drivers for enhancing hybrid rice resistance and dominance [3,4,5]. Globally, China accounted for 50% (15.5 million ha) of the hybrid rice plantation area in 2014, with an average of 6% (6.36 million ha) outside China [5,6]. The CMS/*Rf* system remains promising for hybrid rice production [7]. In China, the prevailing sterile lines in three-line hybrid rice combinations are primarily from the wild-abortive (WA), Honglian (HL), and Boro-Taichung 65 (BT) types (with the Chinsura Boro cytoplasm). In contrast, production-promoted restoration lines of *indica* sterile lines incorporate genetic components from Southeast Asian varieties or lines, with ancestral links to the IR system and exceptional parents such as Minghui 63 and Ce64-7 [8]. This results in a single restoration relationship, posing challenges to realizing hybrid dominance. The utilization of heterosis in rice breeding necessitates exploration of novel fertility restoration genes.

The three primary categories of CMS include wild-abortive (CMS-WA), Honglian (CMS-HL), and Boro-Taichung 65 cytoplasm types (CMS-BT) [2]. D1-cytoplasmic male sterility (CMS-D1) is a sporophytic cytoplasmic sterility type developed from Dongxiang wild rice and exhibits a no-pollen-grain phenotype. Its fertility restoration patterns distinctly differ from other sporophytic sterility types. Functional analysis of CMS-D1 and its candidate gene *orf182* reveals that it induces male sterility by fusing with the mitochondrial transit peptide, resulting in the lack of pollen grain in anthers [9]. The rice CMS-WA sterility gene *WA352* specifically accumulates and interacts with COX11 in the chorionic layer during the microspore mother cell stage. This interaction inhibits the scavenging of reactive oxygen species by COX11, causing ROS accumulation, Cyt c release, and chorionic layer PCD abnormalities, ultimately resulting in pollen sterility in CMS-WA [3,10]. The orfH79, whose expression induces a gametophytic male sterility phenotype, is absent in fertile lines. In CMS-BT sterile lines, the sterility gene orf79 encodes a cytotoxic peptide specifically accumulating in anther microspores, leading to pollen abortion [11,12]. China has cloned rice-fertility-related restoration genes, including *Rf2* in Lead Rice (LD)-type CMS [13], *Rf1a* and *Rf1b*, *Ifr1* in BT-type CMS [14,15], *Rf17* in CW-type CMS [16], *Rf5* and *Rf6* in HL-type CMS [17], Rf4 in WA-type CMS [18], *Rf98* in RT98-type CMS [13], and *Rf19*(t) in WA-type CMS [19]. Aside from *Rf2* and *Rf17*, all other cloned rice *Rf* genes encode pentatricopeptide repeat (PPR) proteins [2]. Nonetheless, in-depth exploration of the molecular mechanisms underlying CMS and fertility restoration in rice is essential.

In various analyses of *Rf* QTLs, over 75 distinct cytoplasmic fertility restoration QTLs were identified across 12 rice chromosomes. Their phenotypic contributions ranged from 2.9% to 61.9%. Although numerous fertility restoration genes from different cytoplasmic sterility types have been found, the majority originate from cultivated and local rice varieties. Research on discovering and localizing restoration genes in wild rice remains limited [19]. Of the 40 localized CMS-WA fertility restoration QTLs, widely distributed on 11 rice chromosomes except chromosome 9 [20,21,22,23,24,25,26,27,28,29,30], 13 were detected on chromosome 1, and on chromosome 10, 1 fertility restoration QTL was pinpointed. Ngangkham et al. [28] located a fertility restoration QTL each on chromosomes 2, 3, 4, 5, and 8. Notably, those on chromosomes 3 and 4, located in *Rfi-3* and *Rfi-4*, contributed over 35% and served as main-effect QTLs. Additionally, nine fertility restoration QTLs were found on chromosomes 6, 7, 11, and 12, all demonstrating micro effects in terms of their genetic effects [20,31]. The CMS-WA fertility restoration primarily depended on two pairs of major fertility restoration genes, each with varying impact. The effects were cumulative and influenced by minor genes. Eight QTLs for restoring fertility in CMS-DA-type cytoplasmic sterility have been located on chromosomes 1, 5, 9, and 10 [32,33,34]. These QTLs were mainly on chromosomes 1 and 10, contributing between 2.9% and 43.2%. CMS-HL sterile cytoplasmcytoplasm, originating from Hainan Red Aawned Wild Rice, represents the typical gametophytic sterility type. Up to now, eight fertility restoration QTLs have been identified, located on chromosomes 1, 8, and 10 [35,36,37,38,39]. In the case of CMS-BT, the four fertility restoration QTLs were localized on rice chromosome 10. Itabashi et al. [13] reported that the restoration gene (*Rf2*) is capable of restoring the fertility of LD-type CMS using a chromosome fragment substitution line. The gene was precisely targeted to the interval RM12939 to RM12955. 

The BIL L5339 exhibits fertility restoration and stress tolerance, enabling the restoration of Dongxiang wild rice’s cytoplasmic sterility type. In this study, a BC_4_F_5_ population was constructed using line L5339 as the material and Xieqingzao B (XB) as the recurrent parent. Phenotypic analysis, focusing on spikelet fertility in crosses with sterile lines, was conducted to pinpoint QTLs associated with fertility restoration in Dongxiang wild rice. The classical QTL mapping method, and new GradedPool-Seq (GPS) approach combining high-throughput sequencing with bulked-segregant analysis (GPS-BSA), was utilized for QTL identification, with subsequent stages focusing on the precise localization and gene cloning. This approach establishes the groundwork for further gene positioning, detailed structural and functional exploration, and analysis of the genetic mechanisms involved in cytoplasmic sterility restoration. Concurrently, the pursuit of new restoration genes in wild rice holds significant practical importance. It contributes to enriching the genetic diversity of restoration genes in hybrid rice, enhancing the utilization of heterosis in rice improvement [40].

## 2. Results

### 2.1. Phenotypic Performance

**Figure 1:**
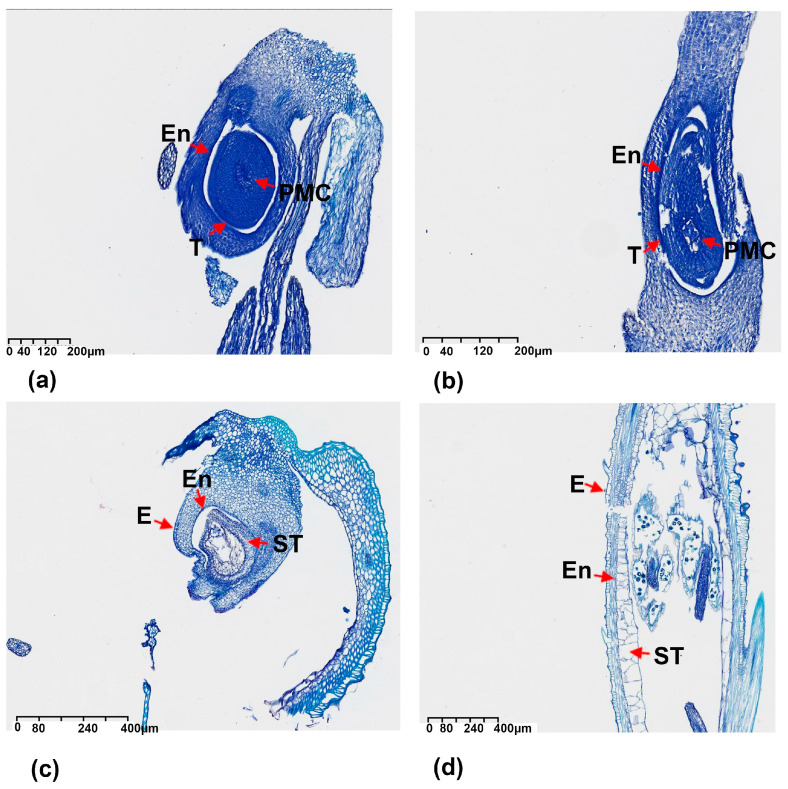
Stamen section of fertile and infertile rice. (**a**,**b**) Fertile rice stamen section from Dongxiang wild rice. (**c**,**d**) Non-fertile rice from DB11A. E: epidermis; En: endothecium; T: tapetal layer; PMC: pollen mother cell; ST: swollen tapetal layer.

The stamen section of fertile and infertile rice is depicted in Figure 1. A summary of the descriptive statistics for spikelet fertility is provided in Table 1. Significant variations in spikelet fertility, exhibiting a continuous distribution, were observed among both tested and F_1_ populations. The F_1_ testcross population planted in 2019 and 2020 exhibited the highest average spikelet fertility (19Z9A/XDX-BIL), followed by a lower two-year average for the Z9A/XDX-BIL population, and even lower spikelet fertility for 20Z9A/XDX-BIL. For the 19Z9A/XDX-BIL population: 6.67–83.66%, mean 36.66%; 20Z9A/XDX-BIL: 5.56–87.46%, mean 34.18%; two-year Z9A/XDX-BIL: 7.33–85.56%, mean 34.63%, surpassing the mean fertility rate of the XB/Z9A F_1_ population (19.03%).

Highly significant (*p* < 0.01) and positive correlations were shown between 19Z9A/XDX-BIL and 20Z9A/XDX-BIL, between 19Z9A/XDX-BIL and two-year average fertility, and between 20Z9A/XDX-BIL and two-year average fertility, with the correlation coefficients of 0.750, 0.934, and 0.943, respectively. These findings confirm the stability of fertility restoration over successive years, validating the accuracy of fruiting rate statistics. Positive correlations (*p* < 0.05) were also evident between the subject populations’ fertility of DB11A/XDX-BIL and Z9A, showing correlation coefficients of 0.188, 0.321, and 0.274, respectively. Notably, skewness and kurtosis values were close to 1, which indicated the influence of multiple genetic loci controlling spikelet fertility as a quantitative trait. This pattern aligns with typical inheritance of quantitative traits, making it suitable for QTL analysis (Table S1).

### 2.2. QTL Localization

**Figure 2:**
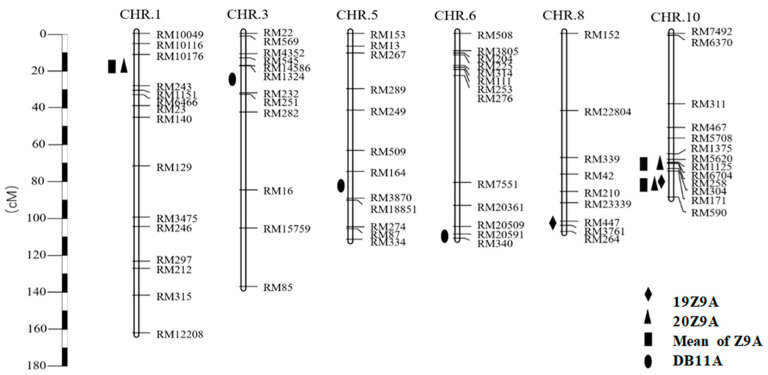
Chromosomal positions of the QTLs conferring fertility restoration for CMS-ID and CMS-DWR.

A total of seven QTLs were detected for fertility restoration of two types of CMS: four for ID-type CMS and three for CMS-type CMS. These QTLs were distributed across rice chromosomes 1, 3, 5, 6, 8, and 10 (Figure 2, Table 2). A single QTL explained phenotypic variance (*R*^2^) ranging from 2.76% to 3.47% for DWR-type CMS and from 3.45% to 12.46% for ID-type CMS.

Regarding ID-type CMS, the Z9A/XDX-BIL population revealed four QTLs for fertility restoration: *qRf1*, *qRf8*, *qRf10.1*, and *qRf10.2*. *qRf1* was detected in the RM10176–RM243 interval on chromosome 1, contributing 4.30% to phenotypic variance in ID-type CMS, with the DWR allele increasing spikelet fertility by 17.47%. *qRf8* was identified in the RM447–RM3761 interval on chromosome 8, contributing 4.99% phenotypic variance and an additive effect value of 16.65%. *qRf10.1* and *qRf10.2* were located in the intervals RM1125–RM6704 and RM5620 and the interval RM171–RM590 on chromosome 10, respectively. *qRf10.1* exhibited an *R*^2^ of 7.04%, with the DWR allele increasing spikelet fertility by 21.99%. *qRf10.2* displayed a phenotypic dedication of 2 greater than 10% in both years of testing, enhancing spikelet fertility by 21.94%. *qRf10.2*, detected for two consecutive years, held the highest LOD value and phenotypic contribution among QTLs in the same year, establishing its role as the primary fertility restoration QTL. Based on the two-year average spikelet fertility, three fertility restoration QTLs were calculated using QTL IciMapping 4.2, aligning with the fertility restoration QTLs detected in 2020.

For DWR-type CMS, the DB11A/XDX-BIL population unveiled three QTLs for fertility restoration: *qRf3*, *qRf5*, and *qRf6*, distributed on chromosomes 3, 5, and 6. *qRf3* and *qRf5* resided in the RM1324–RM232 interval on chromosome 3 and the RM164–RM3870 interval on chromosome 5, respectively. *qRf6* was identified in the RM20591–RM340 interval on chromosome 6. *qRf3* contributed 2.76% to phenotypic variance, with the DWR allele increasing spikelet fertility by 16.56%. *qRf5* contributed 3.47% to phenotypic variance, increasing spikelet fertility by 15.56% with the DWR allele. *qRf6* contributed 3.47% to phenotypic variance, raising spikelet fertility by 15.12%. All QTL favorable alleles originated from Dongxiang wild rice.

### 2.3. GradedPool-Seq (GPS) for qRf3 and qRf6

In this study, the fertility rate of the DB11A/XDX-BIL test population was assessed. Strains exhibiting high, medium, and low fertility were sorted into three distinct phenotypic pools. DNA was extracted from both the parental strains and each phenotypic pool for GPS analysis.

Following high-throughput sequencing, DB11A-H, DB11A-M, and DB11A-L samples were sequenced. Raw data underwent filtration, resulting in a clean read of 1.013 Gbps and clean bases of 152.048 Gbps, with a Q20 of 96.11–96.74% and a 42.01–42.53% GC content (Table 3). Sequenced base distribution appeared normal across all samples (Figure S1). The samples exhibited over 80% matching rate with *indica* rice and over 10% matching rate with japonica rice (except for the parental species). These findings indicate the absence of contamination and adherence to sequencing standards. Based on these results, all samples provided sufficient data volume, confirmed sequencing quality, and displayed normal GC distribution. Comparison between sequencing data and the rice reference genome yielded normal results, enabling subsequent mutation detection and gene localization for future trait analyses (Table S2).

### 2.4. Candidate Genes Associated with Rice Fertility

The Nihon Haru reference genome was used to compare the filtered clean reads of the four samples. The average comparison efficiency with the reference genome stood at 98.19%, while the sequencing depth and genome coverage averaged 96× and 95.48%, respectively. These metrics demonstrated substantial gene coverage and sequencing depth. Analysis revealed fewer heterozygous genotypes in the parents and more in the mixed pool samples, indicating the validity and reliability of SNP results in genome comparison with Haru Nihongo (Tables S3 and S4).

The SNPs data were filtered to retain 1,841,226 SNPs for subsequent analysis. Non-parametric tests were conducted on each SNP locus of the four mixed pools using the Ridit program to calculate statistical *p*-values (genome-wide significance threshold *p* < 5.0 × 10^−8^). Results are presented as *p*-value Manhattan plots prior to applying the noise reduction algorithm.

**Figure 3:**
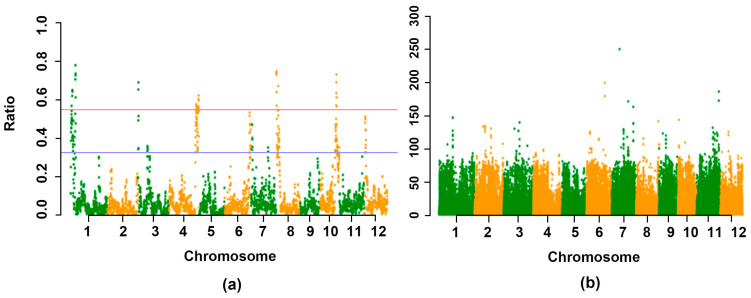
Manhattan figure. Each green and orange dot represents one SNP (**a**) Ridit analyses of the *p*-value Manhattan figure. (**b**) SNP-ratio Manhattan figure. The *p*-value plot is the result of Ridit analysis before the noise-reduction algorithm. The -ln (*p*-value) plot (Y-axis) was plotted against SNP positions (X-axis) on each of the 12 chromosomes. The X-axis value was set at a midpoint at each defined genomic interval, and the Y-axis value corresponds to ratio. The red line indicates the top 1% threshold, and the blue line indicates the top 5% threshold. The horizontal line represents the significance association threshold: the higher the Ration value, the better the effect of association.

While *p*-values were obtained for each variant, they were not sufficient for pinpointing the QTL interval. Thus, a background noise reduction scheme was implemented. This involved calculating the ratio of statistically significant variants to the total within defined intervals. Utilizing a 400 kb window and a 100 kb step, the ratio scatterplot of significant loci to total loci (SNP ratio) was generated. The region with higher peaks, corresponding to the target gene’s location, identified the candidate region for trait association. The ratio plot identified the candidate genetic intervals responsible for the phenotype within the genome, divided into 3702 intervals (Figure 3).

The subsequent results were tabulated for SNP ratio at the top 1% and top 5% thresholds, respectively. Figure 3 displays the chromosome on the horizontal axis, with the threshold of significant association depicted by the horizontal line. A higher ratio value indicates a stronger association effect. The findings revealed that 185 intervals exceeded a SNP ratio of 0.326 at the maximum 5% threshold, and 37 intervals exceeded a SNP ratio of 0.549 at the maximum 1% threshold. This study identified 16 chromosomal regions associated with rice fertility recovery based on the analysis of SNP ratio correlation with the top 5% threshold. These regions were situated on chromosomes 1, 3, 4, 6, 7, 8, 10, and 12 (Table 4). Chromosome 1 exhibited four intervals spanning 2.8 Mb, 0.6 Mb, 0.4 Mb, and 1.1 Mb, correspondingly. Chromosome 3 contained two intervals of 0.9 Mb and 0.8 Mb, correspondingly. The interval on chromosome 4 measured 4.5 Mb. Chromosome 6 displayed two intervals, each measuring 0.9 Mb and 0.8 Mb, correspondingly. Interval sizes were 0.9 Mb and 1.1 Mb, respectively. Additionally, chromosome 7 featured two intervals of sizes 0.8 Mb and 0.5 Mb, while chromosome 8 contained intervals of 1.3 Mb and 2.6 Mb. Chromosome 10 had two intervals measuring 3.3 Mb and 0.3 Mb, while chromosome 12 featured an interval of 1.5 Mb.

For BSA localization, a total of 3752 genes (at the top 5% threshold) were extracted from the candidate intervals. The most significant interval contained 798 genes on chromosome 11, while the least significant interval contained 205 genes on chromosome 7. Among these sixteen candidate regions, eight loci were associated with rice fertility, and five loci were linked to rice grain or grain number.

### 2.5. Correlate Locking qRf3 and qRf6 Physical Distances

A total of 368 molecular markers were used to detect the polymorphism between the parents XB and “Dongxiang wild rice”. Electrophoretic analysis revealed 129 polymorphic SSR markers, representing a 35.1% polymorphism rate between the two parents. These markers were applied for genotyping the XDX-BIL population and subsequent data recording (Figures S2 and S3). Locating the physical positions of the flanked markers of the *qRf3*, *qRf5*, and *qRf6* were conducted using the Gramene website [https://www.gramene.org/ (accessed on 27 September 2023)]. The knowing of whether the flanked intervals of the QTL detected by QTL mapping in alignment with the candidate intervals identified by GPS-BSA was analyzed on the overlapping intervals on chromosomes 3 and 6. For chromosome 3, the fertility restoration locus pinpointed a physical position from 6,036,117 bp to 11,700,000 bp. Therein, *qRf3* ranged from 6,036,117 bp to 9,734,810 bp by the QTL mapping, while being from 10,900,001 bp to 11,700,000 bp by GPS-BSA. Regarding chromosome 6, the fertility restoration locus occupied the interval from 28,016,785 bp to 31,247,556 bp, which was localized by the QTL result analysis to the 28,016,785 bp to 28,599,181 bp range. However, association GPS-BSA analysis located it within the interval from 29,100,001 bp to 31,247,556 bp.

The *qRf3* was detected within the maker interval RM1324-RM232 on chromosome 3 using the DB11A/XDX-BIL population. Its physical region spanned from 6,036,117 bp to 9,734,810 bp. The physical region of *qRf6* flanked by markers RM20591 and RM340 on chromosome 6 ranged from 28,016,785 bp to 28,599,181 bp (Table S5). Two candidate intervals on chromosome 3 were identified through GPS-BSA analysis, with one interval (10,900,001–11,700,000 bp) adjacent to *qRf3*. On chromosome 6, two candidate intervals were located at 29,100,001–30,000,000 bp and 30,100,001–31,247,556 bp, respectively. Notably, the second interval contained genes associated with rice fertility recovery. Not within the GSP-BSA localization candidate region’s top 5%, *qRf5* was excluded as a fertility restoration gene for the “Dongxiang wild rice” sterile line DongB11A.

**Figure 4:**
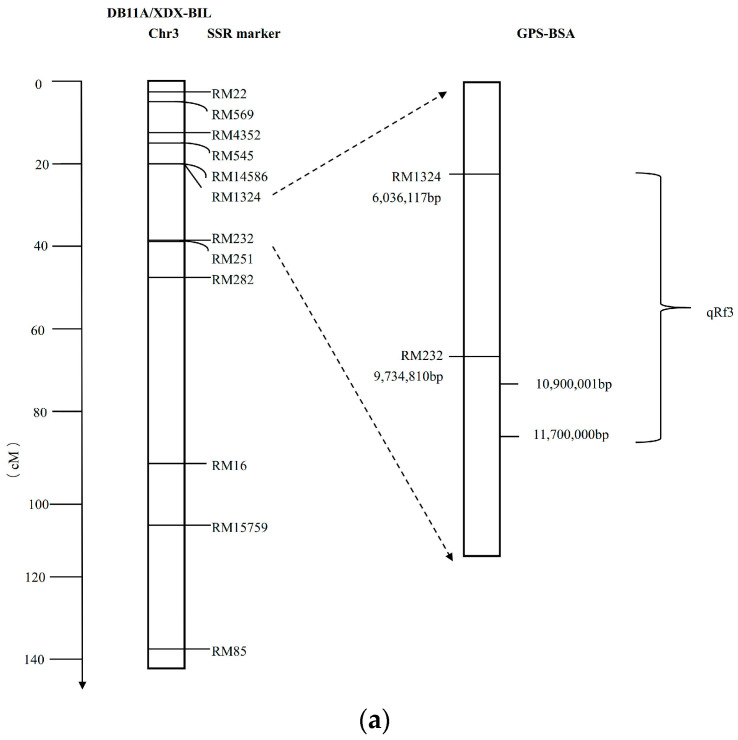

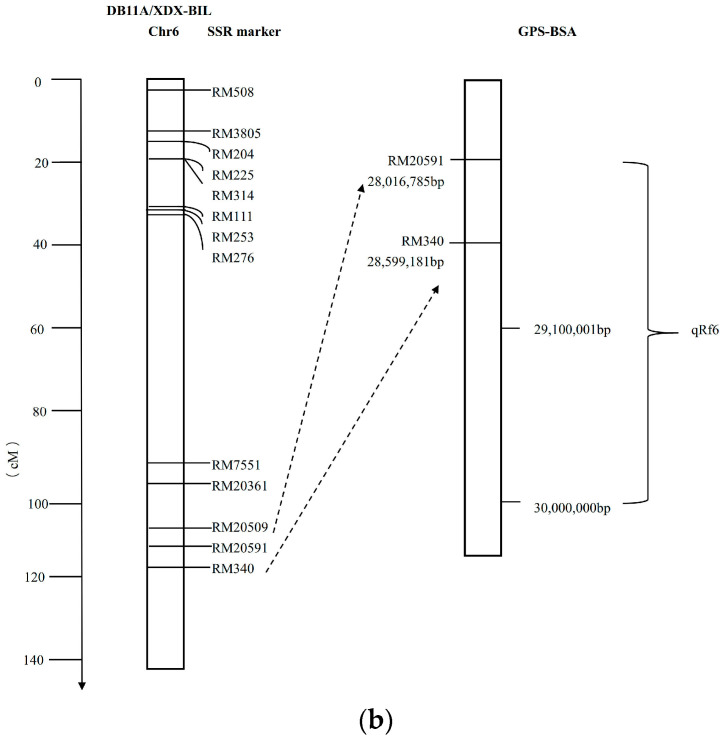
Schematic model of the Rf interval in DB11A/XDX-BIL. (**a**) qRf3 interval. (**b**) qRf6 interval.

In summary, both localization methods pinpointed the fertility recovery locus on chromosome 3 (6,036,117–11,700,000 bp) and chromosome 6 (28,016,785–31,247,556 bp) (Figure 4).

## 3. Discussion

The utilization of hybrid advantage stands as a significant achievement in genetic breeding research for agricultural applications. The restoration gene for cytoplasmic male sterility can revive fertility in rice exhibiting cytoplasmic male sterility. This sterility forms the foundation for harnessing hybrid advantage in three lines of hybrid rice. The successful alignment of the cytoplasmic male sterility and restoration gene system has greatly facilitated the utilization of hybrid advantage, leading to the widespread adoption and application of hybrid rice in China [2]. Research indicates widespread distribution of restoration genes with high frequency in wild rice, while cultivated types [40] exhibit lower frequency. Consequently, the exploration of novel rice repair genes, particularly in wild rice, proves advantageous in breeding new lines of repair rice varieties and advancing the utilization of rice hybrid advantage.

Among the parents employed in this study, “L5339” emerged from a high-generation backcross inbred line developed from the combination of “Dongxiang wild rice” and “Xieqingzao B”. This material possesses phenotypic traits and stress tolerance, and it inherited the cold-tolerant characteristic of “Dongxiang wild rice”, along with exceptional overall characteristics and fertility restoration genes for diverse cytoplasmic sterile lines. Notably, it features fertility restoration genes specific to the cytoplasmic type of sterile lines from “Dongxiang wild rice”, rendering it an optimal choice for “Dongxiang wild rice”. Its particular genetic makeup makes it an excellent candidate for investigating outstanding genes in “Dongxiang wild rice”. In this experiment, four fertility restoration QTLs were identified for the CMS-ID type, and three for the CMS-DWR type, all originating from Dongxiang wild rice. The seven QTLs identified were favorable alleles from “Dongxiang wild rice”. The *Rf* genes of the two sterile lines were non-equivalent. Notably, during production, a series of cytoplasmic sterile lines derived from “Dongxiang wild rice” was developed; however, corresponding restoration sources were absent in existing cultivated rice varieties. This study detected three partial fertility-restoring QTLs sourced from Dongxiang wild rice, forming the basis for the application of Dongxiang wild rice cytoplasmic sterile lines. The findings further underscored the interdependence between cytoplasmic sterility and fertility restoration genes. Restoration genes must be present in wild rice containing sterility genes or within the wild rice population [43]. The high-generation backcross inbred lines, derived from this material, provides a foundation for investigating the genetic mechanisms of other superior genes. This population, along with the parent “L5339”, serves as ideal material for selecting restorer lines. Strengthening the genetic enhancement of this material is essential for its practical application in production.

Researchers in recent years have effectively combined QTL-seq with various techniques to rapidly and accurately map QTLs and genes linked to salt tolerance in gibberella ear rot (GER). Salt tolerance QTL on chromosome 4 of the SR 86 × Nip F_2_ population were swiftly located by Gao et al. [44] using QTL-seq and BSA. Yuan employed a combination of QTL mapping and GPS-seq analysis to identify 29 GPS peak SNPs on chromosome 4 that overlapped with QTL *qGER4.2*, leading to the identification of seven candidate genes associated with GER resistance among the peak SNPs [22]. The novel BSA analysis technique, GPS-seq, localizes QTLs in segregating populations of the target trait. This is achieved by creating distinct DNA mixing pools from offspring individuals with extreme phenotypes, along with parental DNA, and then subjecting the data to sequencing. The Ridit (relative to an identified distribution unit) algorithm calculates allele frequencies for each locus and determines their adherence to the standard distribution, yielding *p*-values. Subsequent noise reduction through the non-overlapping sliding window method refines the *p*-values, achieving QTL localization. Notably, GPS exhibits superior localization accuracy compared to BSA-seq, which relies solely on two extreme phenotypic mixing pools for localization [41]. In our experiment, we applied the QTL localization method in conjunction with GPS-seq to identify seven QTLs associated with fertility restoration in “Dongxiang wild rice”. Among these, three QTLs (*qRf1*, *qRf10.1*, and *qRf10.2*) represent CMS-ID-type fertility restoration and have not been previously reported, while one QTL (*qRf5*) signifies CMS-DWR-type fertility restoration. *qRf1* partially coincides with a fertility restoration QTL reported by Li et al. [42], encompassing the *Rf3* position of CMS-WA-type [16,24]. *qRf10.1* was reported by Li and Hu [32,45], and it aligns with the CMS-WA-type *Rf3* documented by Yao et al. [46]. *qRf10.2* concurs with the fertility QTL intervals from Li et al. [42] and Hu et al. [32], as well as with the *qRf10* interval of the CMS-DA type identified by Xie et al. [33], which is also involved in the recovery of the CMS-WA type of fertility [22,28,47]. The *Rf4* gene encodes a pentapeptide repeat protein, located near *qRf10.2* for CMS-WA type restoration [48]. *qRf5* overlaps with the *qRf5.1* interval reported by Hu et al. [32]. *Rf4* of CMS-WA [26], *Rf5* of CMS-HL [36], *Rf1* of CMS-BT [16], *RfD1(t)* of CMS-Dian1 [47], and *qRf10* of CMS-DA [33], all situated in the same 10th chromosome region, were identified. These alleles were detected in similar chromosomal regions across diverse rice materials, some overlapping with previously reported fertility restoration QTLs. Significance lies in exploring whether these QTLs share gene clusters or functions, as well as in understanding the origin and evolution of fertility restoration genes. This exploration guides the cultivation of restoration lines for new rice varieties, facilitates rice hybrid advantage utilization, and advances genetic breeding research. 

The fertility restoration genes (*qRf3* and *qRf6*) for CMS-DWR line DongB11A were identified on chromosome 3 and 6 through QTL mapping and GPS-BSA analysis, which restore fertility of DongB11A by 16.56% and 15.12%, respectively. These genes are located at physical positions: 6,036,117–11,700,000 bp and 28,016,785–31,247,556 bp, respectively. Few reports exist on fertility restoration QTLs on chromosomes 3 and 6 [25,38], primarily focusing on the CMS-DA and CMS-ID types [25,49]. Hu et al. [25] localized the *qRf3* fertility restoration QTL on chromosome 3, within the RM282-RM16 interval, characterized for CMS-ID type fertility restoration. However, the physical position of this QTL does not overlap with the fertility restoration locus identified in this study on chromosome 3. Similarly, *qRfi-6* and *qRfBSS-6*, QTLs capable of restoring the CMS-WA type, were detected on chromosome 6 by Li et al. [20] and Li et al. [38], respectively. The intervals of these QTL loci overlapped with *qRf6* for CMS-DWR type on chromosome 6. Despite the similarities, the positions of the fertility restoration loci do not overlap, suggesting that these two QTL loci are novel findings for fertility restoration in the “Dongxiang wild rice” sterile line DongB11A. This discovery establishes a foundation for applying and advancing cytoplasmic sterile lines of “Dongxiang wild rice” and introduces new genetic resources for selecting rice restoration lines. The next steps involve candidate gene screening, the construction of complementary and RNAi interference vectors, and *Agrobacterium*-mediated transformation experiments to verify their gene functions and unravel their mechanisms of action.

## 4. Materials and Methods

### 4.1. Plant Materials

**Figure 5:**
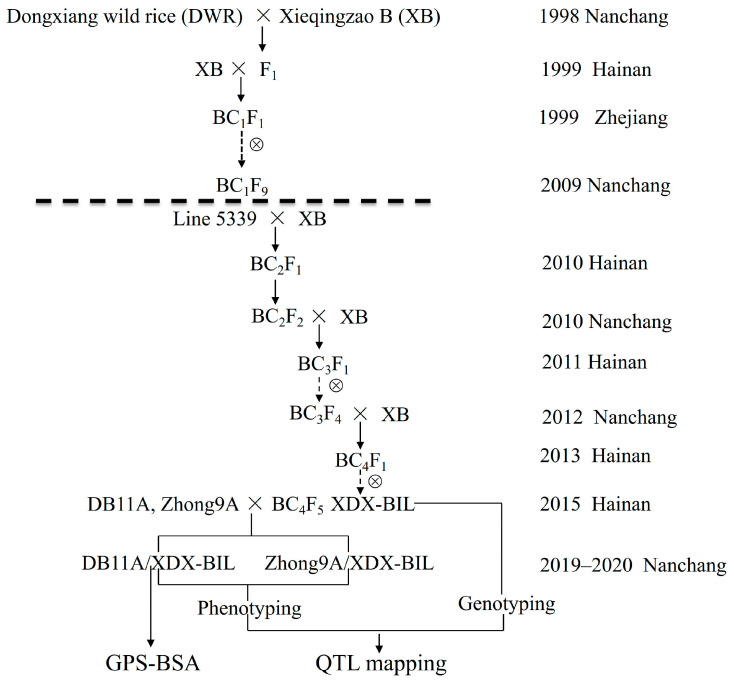
The construction of genetic populations. The arrows represents hybrid offspring and dashed arrows represents self-cross offspring. The “⊗” represents self-cross.

The BC_1_F_10_ backcross inbred lines (BILs) were established by first crossing an accession of Dongxiang wild rice (*Oryza rufipogon* Griff., hereafter referred to as DWR) as the female parent with cultivated Xieqingzao B (hereinafter referred to as “XB”) to generate an F_1_ hybrid in 1998 by the Rice Research Institute, Jiangxi Academy of Agricultural Sciences (RRI, JAAS), Nanchang, China. The recurrent parent XB is the maintainer line of Xieqingzao A belonging to dwarf-wild-abortive-type CMS (*O*. *sativa* L.), and the donor parent DWR is a common wild rice accession collected from in situ conservation populations, which is distributed in northern regions worldwide. Following this, the F_1_ plants were backcrossed to XB to generate a BC_1_F_1_ cross of XB//DWR/XB (hereafter referred to as XDX), from which a BIL population of BC_1_F_10_ lines was obtained by single seed descent in 2008 (Figure 5). Our laboratory selected L5339 from the BC_1_F_10_ XB/DWR/XB BILs based on the results of phenotypic investigation and molecular marker screening in 2009. The single plant, after backcrossing with XB for three times and self-crossing for four times, obtained a stable genetic (XB//DWR/XB) BC_4_F_5_ population (hereafter referred to as XDX-BIL) in Hainan in 2015 by the Rice Research Institute, Jiangxi Academy of Agricultural Sciences (RRI, JAAS), Nanchang, China (Figure 5).

A controlled cross between two alloplasmic CMS lines, including Zhong 9A (Z9A) and DongB11A (DB11A), and each line of the BC_4_F_5_ XDX-BIL population was made, forming two sets of the testcross populations. As a result, a 19Z9A/XDX-BIL cross population was obtained in 2019, and two sets of cross populations and two sets of F_1_ populations (20Z9A/XDX-BIL and DB11A/XDX-BIL, and XB/Z9A and XB/DB11A, respectively) were obtained in 2020. Zhong9A is an *indica* CMS line (*O*. *sativa* L.) with an Indonesia paddy rice cytoplasmic origin (CMS-ID), and DongB11A is an *indica* CMS line (*O*. *sativa* L.) with a DWR cytoplasmic origin (CMS-DWR).

### 4.2. Field Experiments and Phenotyping

During the rice-growing seasons of May to October in both 2019 and 2020, the experiment was conducted in fields at the Rice Research Institute of Jiangxi Academy of Agricultural Sciences, located in Nanchang City, Jiangxi Province, China (latitude: 28°33′ N, longitude: 115°56′ E). In the 2020 experiment, test crosses were performed between the XDX-BIL population and the parental XB using Z9A and DB11A. Over the span of 2019 to 2020, we formed three sets of tested populations and two sets of F_1_ testcross populations: 19Z9A/XDX-BIL, 20Z9A/XDX-BIL, and DB11A/XDX-BIL for the tested populations, and XB/Z9A and XB/DB11A for the F_1_ population. In the field trial, lines were transplanted in rows with an 18 cm plant spacing. Each plot contained five rows with eight plants per row, following standard agricultural practices.

At maturity, five plants were harvested from the central part of each line. In cases of high sterility, only one or two plants were harvested. The remaining test-cross and F_1_ populations were selected randomly from the middle five plants for indoor replication. To assess fertility restoration, we estimated spikelet fertility (SF) as the ratio (percentage) of filled grains to the total grains per panicle. The average SF value from the randomly chosen plants in each line served as the fertility restoration measure for data analysis.

### 4.3. Extraction and Molecular Marker Analysis

Genomic DNA was extracted from fresh field-grown leaves using the Quick and Easy Extraction method. PCR amplification of XB and “Dongxiang wild rice” DNA was conducted with molecular markers to identify polymorphic ones. These polymorphic markers were then utilized for genotype analysis of the tested populations. Amplification reactions consisted of 5 μL FastTaq Premix, 2 μL primer, 1 μL template DNA, and 2 μL ddH_2_O in a 10 µL volume. The amplification process included an initial cycle at 94 °C for 5 min, followed by 35 cycles at 94 °C for 30 s, 55 °C for 30 s, and 72 °C for 45 s. A final extension step was performed at 72 °C for 10 min. Separation of PCR products occurred on a 6% nondenaturing polyacrylamide gel, and bands were visualized through silver staining, based on DNA fragment sizes and band clarity.

### 4.4. Statistical Analysis

Descriptive statistical analysis of spikelet fertility performance in testcross populations and F_1_ plants from XB crosses encompassed range, mean, standard deviation (SD), skewness, kurtosis, and coefficient of variation (CV). SPSS 21.0 software (http://www.spss.com, accessed on 27 September 2023) facilitated these analyses.

### 4.5. Linkage Analysis and QTL Analysis

A linkage map of the BC_4_F_5_ population was constructed using 129 SSRs following Hu et al. [32]. The map spanned 1414.49 centiMorgan (cM) of the 12 rice chromosomes, with an average distance of 10.96 cM between adjacent markers. The linkage map was constructed using Mapmaker/Exp 3.0 software. Distances between markers were estimated using the Kosambi function and presented in cM. QTL analysis was conducted with QTL IciMapping 4.2 using inclusive composite interval mapping of additive (ICIM-ADD). The mapping parameters were fulfilled at the default option of 1.0 cM scanning steps, and 0.01 probability was used in the stepwise regression for additive QTL identification. QTL were verified by a threshold of logarithm of the odds (LOD) scores (2.87–3.32) compared to the threshold calculated from 1000 permutations for *p* < 0.05. Therefore, the marginal threshold for a LOD value more than 2.8 was claimed for the putative QTLs. The analysis encompassed LOD value ranges, as well as additive and phenotypic contributions of each QTL.

### 4.6. GradedPool-Seq (GPS) Sequencing

In the process of identifying genes associated with rice fertility, the DB11A/XDX-BIL testcross population was divided into three ordinal phenotypic pools based on fertility rate: DB11A-H, DB11A-M, and DB11A-L. These pools consisted of 12 strains with high fertility (50–80%), 18 strains with medium fertility (26–36%), and 18 strains with low fertility (6.1–20%). DNA extraction from parental and three distinct fertility rate strains followed an equal-mass mixing principle to ensure consistent DNA concentration. Genomic DNA was extracted from equal masses of fresh leaves (≈0.5 cm) for parental and pooled strains, followed by quality testing. Sequencing was conducted using the Illumina system, and sequencing raw data underwent quality assessment using FASTQC to obtain sequencing quality values (Q-score), AT/GC base content, and base separation. Contamination assessment employed blastn comparison software. GATK mutation analysis software identified potential polymorphic SNP sites across the whole genome, filtered based on quality value, depth, repeatability, etc., resulting in a high credibility SNP dataset. SNP statistics across samples, as well as analysis of genotype numbers and proportions, facilitated comprehensive species understanding. Genomic comparisons and genotyping against reference genome sequences (IRGSP releases build 4.0 pseudomolecules of rice; Os-Nipponbare-Reference IRGSP-1.0; MH 63RS2) were performed using BWA-0.6 software. Screening of parental homozygous genotype SNPs and selection of non-reference bases were carried out from each pool.

Non-parametric tests were executed using the Ridit program to analyze statistical *p*-values for each SNP locus within the four mixed pools, adhering to the genome-wide significance threshold of *p* < 5.0 × 10^−8^. The results display *p*-value Manhattan plots prior to applying the noise reduction algorithm. A scheme for reducing background noise was implemented. The ratio of statistically significant variants to total variants is defined with a 400 kb window and a 100 kb step, the ratio of the number of significant loci to the total number of loci (SNP ratio) was calculated, and the ratio scatterplot was drawn. The region of higher peaks, where the target gene is located, serves as the localization for the candidate trait association. Candidate gene intervals contributing to the phenotype are identifiable using the top 1% and top 5% threshold lines on the ratio plot.

### 4.7. QTL Mapping and GPS-BSA Association Analysis

Fertility restoration loci were identified based on the presence of molecular markers of the QTL within the candidate interval for GPS-BSA localization or overlapping intervals.

## 5. Conclusions

A total of seven QTLs for fertility restoration were mapped on chromosomes 1, 3, 5, 6, 8, and 10. For these QTLs, the enhancing alleles were all from Dongxiang wild rice. Notably, two novel QTLs, *qRf3* and *qRf6*, were located within the interval RM1324–RM232 on chromosome 3 and RM20591–RM340 on chromosome 6, with DWR alleles restoring fertility of DB11A by 16.56% and 15.12%, respectively. Moreover, *qRf10.2* located within the RM171–RM590 interval was stably detected in two years. Meanwhile, *qRf3*, *qRf6*, and *qRf10.2* were also identified by the GPS-BSA method. These findings provide candidates for gene cloning and breeding application of fertility restoration genes from Dongxiang wild rice.

## Figures and Tables

**Table 1:** The spikelet fertility performance of testcross populations and F_1_ plants from Xieqingzao B (XB) crossed with CMS lines.

Testcross Populations	SD	Mean (%)	CV	Skewness	Kurtosis	Range (%)	F_1_ Fertility ^a^ (%)
19Z9A/XDX-BIL	23.31	36.66	0.64	−1.16	0.45	6.67–83.66	-
20Z9A/XDX-BIL	26.06	34.18	0.76	−1.29	0.60	5.56–87.46	19.03
The two-year average fertility rate	22.94	34.63	0.66	−0.86	0.70	7.33–85.56	-
DB11A/XDX-BIL	17.28	30.75	0.56	−0.54	0.57	6.41–68.22	20.03

Note: ^a^ F_1_ fertility means the average F_1_ fertility derived from parent XB and CMS lines (Z9A and DB11A).

**Table 2:** QTLs conferring fertility restoration for CMS-ID and CMS-DWR detected in the XB//DWR/XB BIL populations.

Population	Year	Chr.	QTL	Interval	LOD	Additive Effect	The Proportion of the Variance Explained (%)	References
Z9A/XDX-BILs (CMS-ID)	2019	chr08	*qRf8*	RM447–RM3761	2.83	16.65	4.99	-
		chr10	*qRf10.2*	RM171–RM590	7.45	21.27	10.29	[41,42]
	2020	chr01	*qRf1*	RM10176–RM243	3.35	17.47	4.30	[42]
		chr10	*qRf10.1*	RM1125–RM6704	6.46	21.99	7.04	[41,42]
		chr10	*qRf10.2*	RM171–RM590	11.60	23.36	12.17	[41,42]
	Mean ^a^	chr01	*qRf1*	RM10176–RM243	3.05	21.36	3.45	[42]
		chr10	*qRf10.1*	RM1125–RM6704	7.59	17.32	5.49	[41,42]
		chr10	*qRf10.2*	RM171–RM590	14.79	21.94	12.46	[41,42]
DB11A/XDX-BILs (CMS-DWR)	2020	chr03	*qRf3*	RM1324–RM232	2.84	16.56	2.76	This study
		chr05	*qRf5*	RM164–RM3870	3.01	15.56	3.47	[42]
		chr06	*qRf6*	RM20591–RM340	3.72	15.12	3.47	This study

Note: Additive effect of replacing a Xieqingzao B allele by Dongxiang wild rice allele. ^a^ Mean of spikelet fertility in the years 2019 and 2020.

**Table 3:** Summary of sequencing data quality.

Sample	Clean Reads	Clean Bases	Read Length (bp)	Q20 (%)	GC (%)
DB11A-L	345,694,258	51,854,138,700	150	96.74%	42.48%
DB11A-M	313,883,428	47,082,514,200	150	96.66%	42.53%
DB11A-H	283,917,660	42,587,649,000	150	96.62%	42.01%
XB	70,171,138	10,525,670,700	150	96.11%	42.08%

Clean Bases = Clean Reads × Read length.

**Table 4:** Candidate region statistics for GPS-BSA (top 5%).

Chr.	Start (bp)	End (bp)	Symbol Gene	Gene Annotation
chr01	300,001	3,100,000	*SL1|OsJAG* (*Os01g0129200*)	C_2_H_2_ zinc-finger transcription factor
chr01	3,200,001	3,800,000	*FIB|TSG1* (*Os01g0169800*)	Tryptophan aminotransferase
chr01	4,500,001	5,000,000	*GW5L* (*Os01g0190500*)	IQ domain-containing protein
chr01	5,200,001	6,300,000	*OSMADS3* (*Os01g0201700*)	MADS-box family gene
chr03	200,001	1,100,000	*OsDRM2*(*Os03g0110800*) *PAIR1* (*Os03g0106300*) *OSHB1|LF1* (*Os03g0109400*)	DNA methyltransferase Coiled-coil protein Class III homeodomain-leucine zipper protein
chr03	10,900,001	11,700,000	*OsNAS2* (*Os03g0307200*) *OsPME1* (*Os03g0309400*)	Nicotianamine synthase 2 Pectin methyl esterase
chr04	30,900,001	35,400,000	*MOC3*|*OsWUS*|*TAB1*|*OsTAB1* (*Os04g0663600*) *OsAAE3* (*Os04g0683700*) *OsAGO2* (*Os04g0615700*) *OsCP1* (*Os04g0670500*) *OsCER2*|*OsHMS1I* (*Os04g0611200*)	Homeobox protein orthologous to *Arabidopsis* WUS Acyl-activating enzyme 3 ARGONAUTE (AGO) family protein Cysteine protease Cofactor of beta-ketoacyl-CoA synthase
chr06	29,100,001	30,000,000	*OsPTR9|OsNPF8.20* (*Os06g0706400*)	PTR/NRT1 family protein
chr06	30,100,001	31,247,556	*OsPMT16* (*Os06g0712800*)	Pectin methyltransferase
chr07	1,200,001	2,000,000	*OsAAP1* (*Os07g0134000*)	Amino acid permease
chr07	19,900,001	20,400,000	-	-
chr08	1	1,300,000	*PSS1* (*Os08g0117000*)	Kinesin-1-like protein
chr08	1,400,001	4,000,000	*CYP703A3* (*Os08g0131100*) *OsTPL|ASP1|OsLIS-L1* (*Os08g0162100*)	Cytochrome P450 hydroxylase Transcriptional co-repressor
chr10	17,900,001	21,200,000	*OsABCG26* (*Os10g0494300*) *OsNP1* (*Os10g0524500*)	ATP binding cassette G transporter Glucose-methanol-choline oxidoreductase
chr10	22,900,001	23,205,864	*JMJ706|OsJMJ706* (*Os10g0577600*)	H3K9 demethylase
chr12	1,000,001	2,500,000	*ONAC300* (*Os12g0123800*) *OsPIN1d* (*Os12g0133800*)	NAM protein domain containing protein PIN protein

## Data Availability

The data presented in this study are available on request from the corresponding author.

## Supplementary Materials

The following supporting information can be downloaded at: https://www.mdpi.com/article/10.3390/ijms241914832/s1.
