# Supplementary material for: Genetic Analysis of Novel Fertility Restoration Genes (qRf3 and qRf6) in Dongxiang Wild Rice Using GradedPool-Seq Mapping and QTL-Seq Correlation Analysis
Source: Int J Mol Sci. 2023 Oct 2;24(19):14832. doi: 10.3390/ijms241914832 (PMC10573815; doi:10.3390/ijms241914832)
Supplement: Supplementary file 1 [file ijms-24-14832-s001.zip › Supplementary Table S1.pdf]

Table S1 Correlation analysis of spikelet fertility between testcross populations

**Cross-testing groups**	**19Z9A/XDX-BIL**	**20Z9A/XDX-BIL**	**Two-year average bearing rate**	**DB11A/XDX-BIL**
19Z9A/XDX-BIL	1	0.750**	0.934**	0.188
20Z9A/XDX-BIL		1	0.943**	0.321*
The two-year average fertility rate			1	0.274*
DB11A/XDX-BIL				1

Note： * , ** significant at 0.05 and 0.01 level, respectively.
