# Supplementary material for: Genetic Analysis of Novel Fertility Restoration Genes (qRf3 and qRf6) in Dongxiang Wild Rice Using GradedPool-Seq Mapping and QTL-Seq Correlation Analysis
Source: Int J Mol Sci. 2023 Oct 2;24(19):14832. doi: 10.3390/ijms241914832 (PMC10573815; doi:10.3390/ijms241914832)
Supplement: Supplementary file 1 [file ijms-24-14832-s001.zip › Supplementary Table S2.pdf]

Table S2. Results of sequencing data contamination test

**Sample**	**Top1 species (comparison rate)**	**Top2 species (comparison rate)**
DB11A-H	*Oryza sativa* Indica (80.92%)	*Oryza sativa* Japonica (10.47%)
DB11A-L	*Oryza sativa* Indica (81.37%)	*Oryza sativa* Japonica (10.79%)
DB11A-M	*Oryza sativa* Indica (81.23%)	*Oryza sativa* Japonica (10.53%)
XB	*Oryza sativa* Indica (84.93%)	*Oryza sativa* Japonica (6.74%)
