# Supplementary material for: Genetic Analysis of Novel Fertility Restoration Genes (qRf3 and qRf6) in Dongxiang Wild Rice Using GradedPool-Seq Mapping and QTL-Seq Correlation Analysis
Source: Int J Mol Sci. 2023 Oct 2;24(19):14832. doi: 10.3390/ijms241914832 (PMC10573815; doi:10.3390/ijms241914832)
Supplement: Supplementary file 1 [file ijms-24-14832-s001.zip › Supplementary Table S3.pdf]

Table S3. Result of genome comparison with Nipponbare

Sample	Mapped Reads Ratio	Coverage	Depth
DB11A-L	98.00%	96.16%	106
DB11A-M	98.46%	96.67%	129
DB11A-H	98.01%	96.99%	118
XB	98.28%	92.10%	29
