# Supplementary material for: Genetic Analysis of Novel Fertility Restoration Genes (qRf3 and qRf6) in Dongxiang Wild Rice Using GradedPool-Seq Mapping and QTL-Seq Correlation Analysis
Source: Int J Mol Sci. 2023 Oct 2;24(19):14832. doi: 10.3390/ijms241914832 (PMC10573815; doi:10.3390/ijms241914832)
Supplement: Supplementary file 1 [file ijms-24-14832-s001.zip › Supplementary Table S4.pdf]

Table S4. Genotype number statistics of each sample

Sample	count	SNP count	Hom AA count	Het RA count	Hom RR count
DB11A-L	3,721,415	3,482,583	1,807,153	1,675,430	238,832
DB11A-M	3,739,132	3,506,071	1,785,665	1,720,406	233,061
DB11A-H	3,748,286	3,539,552	1,787,332	1,752,220	208,734
XB	3,015,680	2,513,461	502,219	506,067	3,015,680
