# Supplementary material for: Genetic Analysis of Novel Fertility Restoration Genes (qRf3 and qRf6) in Dongxiang Wild Rice Using GradedPool-Seq Mapping and QTL-Seq Correlation Analysis
Source: Int J Mol Sci. 2023 Oct 2;24(19):14832. doi: 10.3390/ijms241914832 (PMC10573815; doi:10.3390/ijms241914832)
Supplement: Supplementary file 1 [file ijms-24-14832-s001.zip › Supplementary Table S5.pdf]

Table S5. SSR molecular marker details table.

Chromosome	Marker name	Physical location	Primer forward(5'→3')	Primer reverse(3'→5')
Chr.1	RM10049	746,085	attgacaattgttgaagcgagaaaaggg	cctccagggtcttcgaccgccagt
Chr.1	RM10116	2,169,138	atcccacttgcagacgtagg	acagcagcaaggtgcctc
Chr.1	RM10176	3,637,718	agcaccatcagctcagctactcc	gaaggcttctgtcaagacgatcc
Chr.1	RM243	7,970,722	gatctgcagactgcagttgc	agctgcaacgatgttgtcc
Chr.1	RM1151	8,577,052	gaccgcaaaagatcatcgac	gtaacagcgaccgttggttg
Chr.1	RM6466	9,191,088	cgaacgagaactccctcatg	attgcaccaagaggagatcg
Chr.1	RM23	10,705,594	cattggagtggaggctgg	gtcaggcttctgccattctc
Chr.1	RM140	12,300,716	tgcctcttccctggctcccctg	ggcatgccgaatgaaatgcatg
Chr.1	RM129	19,008,692	tctctccggagccaaggcgagg	cgagccacgacgcgatgtaccc
Chr.1	RM3475	26,041,024	gtcggtttgcctagttgagc	ttcctcggtgtatgggtctc
Chr.1	RM246	27,335,176	gagctccatcagccattcag	ctgagtgctgctgcgact
Chr.1	RM297	32,099,566	tctttggaggcgagctgag	cgaagggtacatctgcttag
Chr.1	RM212	33,053,493	ccactttcagctactaccag	cacccatttgtctctcattatg
Chr.1	RM315	36,734,135	gaggtacttcctccgtttcac	agtcagctcactgtgcagtg
Chr.1	RM12208	41,941,608	gggaggcatgtagatgcagagc	ctccaatggtgaaatcgaatgg
Chr.2	RM154	1,083,820	gacggtgacgcactttatgaacc	cgatctgcgagaaaccctctcc
Chr.2	RM1285	3,837,901	tctccagggacagggtacac	gtggtacaatgtgcagtggc
Chr.2	RM3294	5,204,941	ttacacacactacggacgcg	cctggtggtacctctcttaatc
Chr.2	RM5780	5,453,144	gctgctgcatcttctactgc	acgcacatgcctaagcctag
Chr.2	RM71	8,760,433	ctagaggcgaaaacgagatg	gggtgggcgaggtaataatg
Chr.2	RM29	16,575,205	cagggacccacctgtcatac	aacgttggtcatatcggtgg
Chr.2	RM475	20,399,319	cctcacgattttcctccaac	acggtgggattagactgtgc
Chr.2	RM262	20,794,972	cattccgtctcggctcaact	cagagcaaggtggcttgc
Chr.2	RM6	29,579,845	gtcccctccacccaattc	tcgtctactgttggctgcac
Chr.2	RM213	34,652,316	atctgtttgcaggggacaag	aggtctagacgatgtcgtga
Chr.2	RM208	35,135,783	tctgcaagccttgtctgatg	taagtcgatcattgtgtggacc
Chr.2	RM138	35,674,147	agcgcaacaaccaatccatccg	aagaagctgcctttgacgctatgg
Chr.3	RM22	1,519,587	ggtttgggagcccataatct	ctgggcttctttcactcgtc
Chr.3	RM569	1,888,272	gacattctcgcttgctcctc	tgtcccctctaaaaccctcc
Chr.3	RM4352	4,313,896	gttgttgcaccatagtcaga	atacattcatgaaacctgcc
Chr.3	RM545	4,947,911	caatggcagagacccaaaag	ctggcatgtaacgacagtgg
Chr.3	RM14586	5,987,554	ggtggtgagccaagaactgacc	cctcaaggaatcctcgtaagtcg

Chr.3	RM1324	6,036,117	tgttgatccccttgataggg	agcaagatcagctagctgcc
Chr.3	RM232	9,734,810	ccggtatccttcgatattgc	ccgacttttcctcctgacg
Chr.3	RM251	9,928,907	gaatggcaatggcgctag	atgcggttcaagattcgatc
Chr.3	RM282	12,407,382	ctgtgtcgaaaggctgcac	cagtcctgtgttgcagcaag
Chr.3	RM16	23,126,064	cgctagggcagcatctaaa	aacacagcaggtacgcgc
Chr.3	RM15759	28,314,615	caggatcggacaggatcacagg	gctcctggcgcagctatagacc
Chr.3	RM85	36,348,226	ccaaagatgaaacctggattg	gcacaaggtgagcagtcc
Chr.4	RM335	688,353	gtacacacccacatcgagaag	gctctatgcgagtatccatgg
Chr.4	RM518	2,030,135	ctcttcactcactcaccatgg	atccatctggagcaagcaac
Chr.4	RM307	13,126,513	gtactaccgacctaccgttcac	ctgctatgcatgaactgctc
Chr.4	RM401	13,154,172	tggaacagatagggtgtaaggg	ccgttcacaacactatacaagc
Chr.4	RM6314	18,444,943	gattcgtgtcggttgtcaag	ggttcagggacgaatttcag
Chr.4	RM471	18,824,746	acgcacaagcagatgatgag	gggagaagacgaatgtttgc
Chr.4	RM273	24,044,220	gaagccgtcgtgaagttacc	gtttcctacctgatcgcgac
Chr.4	RM241	26,857,374	gagccaaataagatcgctga	tgcaagcagcagatttagtg
Chr.4	RM280	34,989,558	acacgatccactttgcgc	tgtgtcttgagcagccagg
Chr.5	RM153	189,782	gcctcgagcatcatcatcag	atcaacctgcacttgcctgg
Chr.5	RM13	1,989,212	tccaacatggcaagagagag	ggtggcattcgattccag
Chr.5	RM267	2,881,317	tgcagacatagagaaggaagtg	agcaacagcacaacttgatg
Chr.5	RM289	7,807,745	ttccatggcacacaagcc	ctgtgcacgaacttccaaag
Chr.5	RM249	10,776,494	ggcgtaaaggttttgcatgt	atgatgccatgaaggtcagc
Chr.5	RM509	16,324,561	tagtgagggagtggaaacgg	atcgtccccacaatctcatc
Chr.5	RM164	19,196,472	tcttgcccgtcactgcagatatcc	gcagccctaatgctacaattcttc
Chr.5	RM3870	22,900,326	tacatctccggcgtttacac	ccaaggttgaaacaggaagc
Chr.5	RM18851	23,154,908	gcatcgttcaggtgatttaccc	ggacgagttagtaagcacgatgg
Chr.5	RM274	26,848,154	cctcgcttatgagagcttcg	cttctccatcactcccatgg
Chr.5	RM87	27,104,235	cctctccgatacaccgtatg	gcgaaggtacgaaaggaaag
Chr.5	RM334	28,547,534	gttcagtgttcagtgccacc	gactttgatctttggtggacg
Chr.6	RM508	441,616	ggatagatcatgtgtgggggg	acccgtgaaccacaaagaac
Chr.6	RM3805	2,853,061	agaggaagaagccaaggagg	catcaacgtaccaaccatgg
Chr.6	RM204	3,168,314	gtgactgacttggtcataggg	gctagccatgctctcgtacc
Chr.6	RM225	3,416,533	tgcccatatggtctggatg	gaaagtggatcaggaaggc
Chr.6	RM314	4,878,802	ctagcaggaactcctttcagg	aacattccacacacacacgc
Chr.6	RM111	5,096,744	cacaacctttgagcaccgggtc	acgcctgcagcttgatcaccgg
Chr.6	RM253	5,425,408	tccttcaagagtgcaaaacc	gcattgtcatgtcgaagcc

Chr.6	RM276	6,230,045	ctcaacgttgacacctcgtg	tcctccatcgagcagtatca
Chr.6	RM7551	20,948,206	tcacctccttctgccatctc	ctaactcacctccagcctgc
Chr.6	RM20361	24,099,497	cttgaaatttgtgcggaggttgc	gatgtcaccatcacggagaattagg
Chr.6	RM20509	27,027,550	caccgtcggagaaggactactgg	gagcagccgtaggtgtcaaatgc
Chr.6	RM20591	28,016,785	tcgtctgcgcgaatatttagagagg	atctgcatcggagtcagcaacg
Chr.6	RM340	28,599,181	ggtaaatggacaatcctatggc	gacaaatataagggcagtgtgc
Chr.7	RM5752	2,566,819	ttgcaattaattcgatctcc	gcagatcgattcgttagttc
Chr.7	RM481	2,874,465	tagctagccgattgaatggc	ctccacctcctatgttgttg
Chr.7	RM320	18,693,223	caacgtgatcgaggatagatc	ggatttgcttaccacagctc
Chr.7	RM11	19,256,914	tctcctcttcccccgatc	atagcgggcgaggcttag
Chr.7	RM346	21,043,408	cgagagagcccataactacg	acaagacgacgaggagggac
Chr.7	RM336	21,871,205	cttacagagaaacggcatcg	gctggtttgtttcaggttcg
Chr.7	RM18	25,652,511	ttccctctcatgagctccat	gagtgcctggcgctgtac
Chr.7	RM3555	27,890,587	tggaagtttcctggcgatag	tggttggactgaaaagtccc
Chr.8	RM152	682,963	gaaaccaccacacctcaccg	ccgtagaccttcttgaagtag
Chr.8	RM22804	11,313,822	ggaccaacctaagcagtgactcg	ctgaagagcgatcatcaaatgtgg
Chr.8	RM339	17,812,339	gtaatcgatgctgtgggaag	gagtcatgtgatagccgatatg
Chr.8	RM42	20,094,533	atcctaccgctgaccatgag	tttggtctacgtggcgtaca
Chr.8	RM210	22,471,837	tcacattcggtggcattg	cgaggatggttgttcacttg
Chr.8	RM23339	24,012,302	acagcctatagctcacaccaaacc	gaacacctccgtctccattgc
Chr.8	RM447	26,546,992	cccttgtgctgtctcctctc	acgggcttcttctccttctc
Chr.8	RM3761	27,106,702	cctcaacaatagcaccaccc	ctgcaagtctgcaagcacag
Chr.8	RM264	27,926,632	gttgcgtcctactgctacttc	gatccgtgtcgatgattagc
Chr.9	RM444	5,925,016	gctccacctgcttaagcatc	tgaagaccatgttctgcagg
Chr.9	RM219	7,888,320	cgtcggatgatgtaaagcct	catatcggcattcgcctg
Chr.9	RM1896	11,767,349	ggacagggtaaagtgttaga	cctaagacctatcaactcca
Chr.9	RM278	19,320,020	gtagtgagcctaacaataatc	tcaactcagcatctctgtcc
Chr.9	RM107	20,068,688	agatcgaagcatcgcgcccgag	actgcgtcctctgggttcccgg
Chr.9	RM201	20,174,289	ctcgtttattacctacagtacc	ctacctcctttctagaccgata
Chr.9	RM205	22,720,624	ctggttctgtatgggagcag	ctggcccttcacgtttcagtg
Chr.10	RM7492	53,666	agatggttgccaagagcatg	gtcacgtggcgatttaggag
Chr.10	RM6370	344,385	ttgacaagccacacacacag	gtcctcccttggttctttcc
Chr.10	RM311	9,747,442	tggtagtataggtactaaacat	tcctatacacatacaaacatac
Chr.10	RM467	13,488,471	ggtctctctctctctctctctc	ctcctgacaattcaactgcg
Chr.10	RM5708	14,456,288	aattagaaccatctgaattg	gtatttaaggtcacgtatcg

Chr.10	RM1375	16,644,899	ctacacgcgcaaactctgtc	atgaaggtctaggctgcacc
Chr.10	RM5620	17,403,621	tcgacttgaagcatcacacc	tctgaaatgtcaagtgggcc
Chr.10	RM1125	17,771,479	ggggccagagttttcttcag	gtacgcgcagaaaatgagag
Chr.10	RM6704	17,934,263	cacacattgcattacgaggg	caggggcagcttgaatactg
Chr.10	RM258	18,014,265	tgctgtatgtagctcgcacc	tggcctttaaagctgtcgc
Chr.10	RM304	18,655,588	tcaaaccggcacatataagac	gatagggagctgaaggagatg
Chr.10	RM171	19,048,795	aacgcgaggacacgtacttac	acgagatacgtacgcctttg
Chr.10	RM590	23,043,156	catctccgctctccatgc	ggagttggggtcttgttcg
Chr.11	RM286	383,711	ggcttcatctttggcgac	ccggattcacgagataaactc
Chr.11	RM332	2,840,211	gcgaaggcgaaggtgaag	catgagtgatctcactcaccc
Chr.11	RM167	4,073,024	gatccagcgtgaggaacacgt	agtccgaccacaaggtgcgttgtc
Chr.11	RM202	9,001,608	cagattggagatgaagtcctcc	ccagcaagcatgtcaatgta
Chr.11	RM287	16,767,319	ttccctgttaagagagaaatc	gtgtatttggtgaaagcaac
Chr.11	RM21	19,137,833	acagtattccgtaggcacgg	gctccatgagggtggtagag
Chr.11	RM206	22,014,679	cccatgcgtttaactattct	cgttccatcgatccgtatgg
Chr.11	RM254	23,729,167	agccccgaataaatccacct	ctggaggagcatttggtagc
Chr.11	RM1233	26,534,048	ttcgttttccttggttagtg	attggctcctgaagaagg
Chr.11	RM224	27,673,353	atcgatcgatcttcacgagg	tgctataaaaggcattcggg
Chr.11	RM144	28,281,693	tgccctggcgcaaatttgatcc	gctagaggagatcagatggtagtgcatg
Chr.12	RM20A	970,538	atcttgtccctgcaggtcat	gaaacagaggcacatttcattg
Chr.12	RM19	2,432,080	caaaaacagagcagatgac	ctcaagatggacgccaaga
Chr.12	RM247	3,185,384	tagtgccgatcgatgtaacg	catatggttttgacaaagcg
Chr.12	RM101	8,826,555	gtgaatggtcaagtgacttaggtggc	acacaacatgttccctcccatgc
Chr.12	RM28339	20,742,171	gttcaagtcgatgcggctctgg	cgacgagaagaggtcgcagagg
Chr.12	RM270	25,002,547	ggccgttggttctaaaatc	tgcgcagtatcatcggcgag
Chr.12	RM2734	26,153,565	gctctactgctctagagcaa	gccacggattaatatatgaa
Chr.12	RM17	26,954,657	tgccctgttattttcttctctc	ggtgatcctttcccatttca
