# Supplementary figures and images for: Genetic Analysis of Novel Fertility Restoration Genes (qRf3 and qRf6) in Dongxiang Wild Rice Using GradedPool-Seq Mapping and QTL-Seq Correlation Analysis
Source: Int J Mol Sci. 2023 Oct 2;24(19):14832. doi: 10.3390/ijms241914832 (PMC10573815; doi:10.3390/ijms241914832)

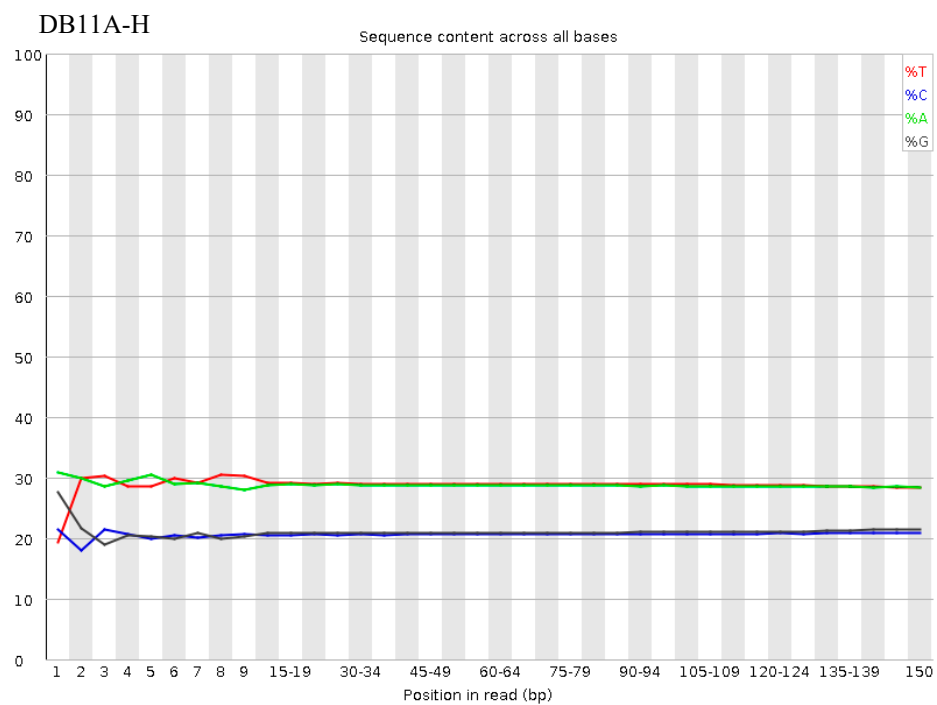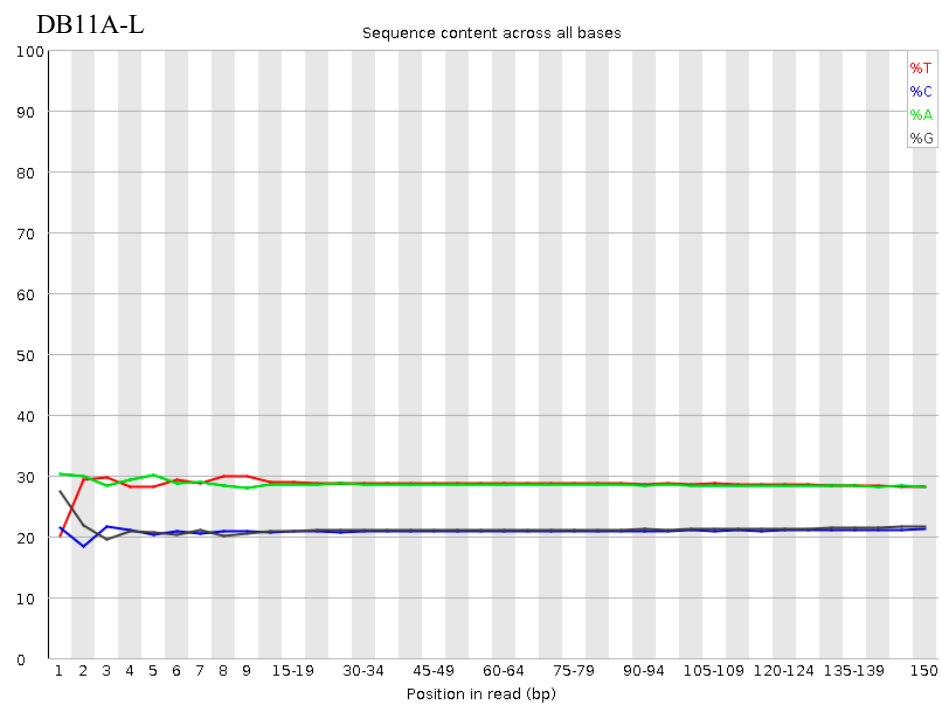

DB11A-M

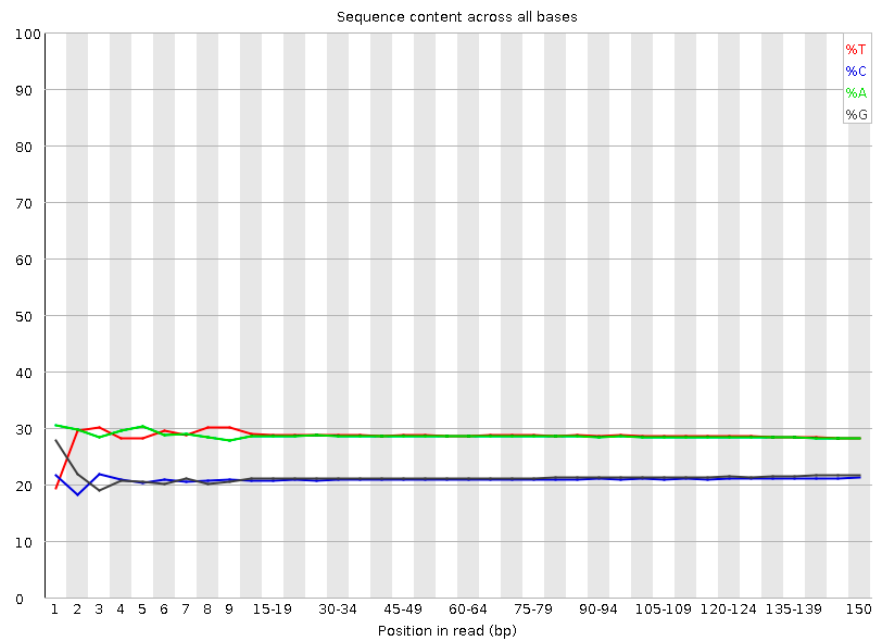

XB

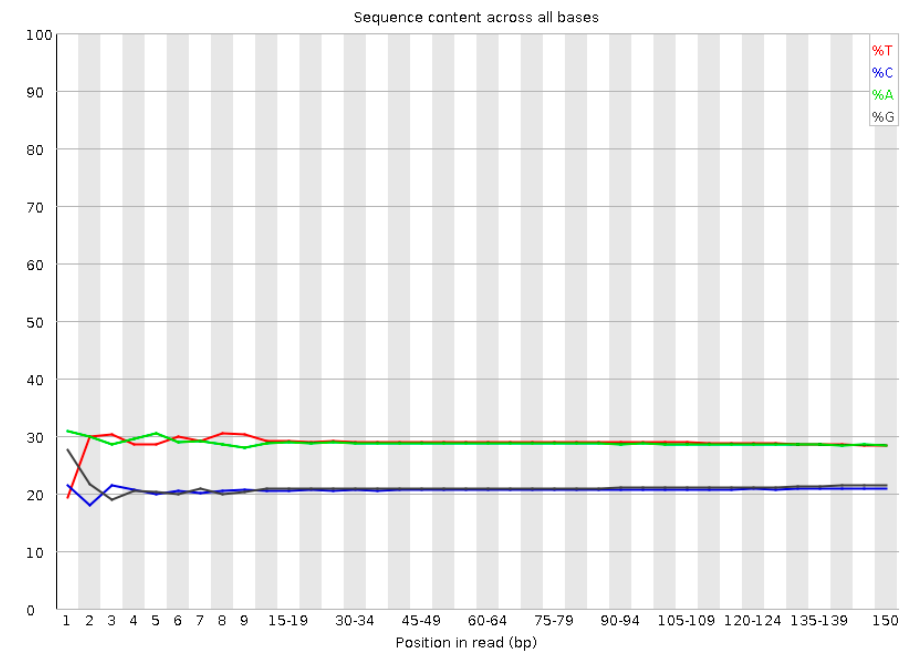

**Figure S1.** The distribution of sequencing bases in each sample.

Supplement: Supplementary file 1 [file ijms-24-14832-s001.zip › Supplementary Figure S1.pdf]
